# Challenges and Opportunities in Characterizing the Genetics of Stuttering: From Sample Acquisition to Functional Interpretation of the Genome

**DOI:** 10.1044/2025_JSLHR-25-00093

**Published:** 2025-10-17

**Authors:** Dillon G. Pruett, Alyssa C. Scartozzi, Hannah G. Polikowsky, Heather M. Highland, Doug M. Shaw, Lauren E. Petty, Alex S. Petty, Shelly Jo Kraft, Jennifer E. Below

**Affiliations:** a Vanderbilt Genetics Institute, Vanderbilt University Medical Center, Nashville, TN; b Department of Epidemiology, Gillings School of Global Public Health, University of North Carolina at Chapel Hill; c Department of Communication Sciences and Disorders, Wayne State University, Detroit, MI

## Abstract

**Purpose:**

Converging etiological evidence supports a genetic risk for developmental stuttering; however, major gaps detailing the genetic architecture remain. Technological advances in genetics have allowed us to explore novel approaches to analyzing this complex trait, but conducting robust and replicable genetic studies requires large, well-phenotyped cohorts of subjects. This article reviews previous research strategies employed to overcome these challenges in identifying genetic variants associated with stuttering and translating stuttering-associated variants into molecular and cellular mechanisms.

**Method:**

We present an overview of data sources and strategies research teams have utilized for the genetic study of stuttering, highlighting the advantages and limitations of each approach. Primary data sources include (a) the International Stuttering Project, (b) the National Longitudinal Study of Adolescent to Adult Health, (c) BioVU, and (d) 23andMe, Inc. In addition to genome-wide association studies (GWASs), we review multiple post-GWAS follow-up analyses to probe the functional impact of stuttering-associated genetic variants and offer new transcriptome-wide analyses.

**Results:**

To date, a diverse array of approaches has resulted in the identification of over 50 stuttering-associated genes. Many genetic associations were near or within genes previously linked to known neurological traits, highlighting a neurological role in stuttering. Additionally, validation studies using polygenic risk scores suggested a high level of genetic concordance between our samples. Functional follow-up studies suggest stuttering-associated variants may affect gene expression in tissues relevant to speech-related structures and neural correlates.

**Conclusions:**

While understanding how specific regions of the genome contribute to stuttering risk remains complex, research from our team and others has utilized a variety of data sources in an attempt to overcome previous limitations in the identification of genetic variation associated with stuttering. As the field of genetics evolves toward large-scale biobanks for research and discovery, prioritizing inclusion of traits such as stuttering will be key.

**Supplemental Material:**

https://doi.org/10.23641/asha.30299764

Genetics play a role in a range of speech, language, and communication traits (Doust et al., 2022; Eising et al., 2022; Lai et al., 2001; Miscimarra et al., 2007; Pariani et al., 2009; Polikowsky et al., 2021; Stein et al., 2004). For stuttering, a condition marked by repetitions, prolongations, and blocks during speech, multiple lines of evidence suggest a large genetic risk profile (Ambrose et al., 1993; Domingues et al., 2014; Dworzynski et al., 2007; Fagnani et al., 2011; Mohammadi et al., 2017; Morgan et al., 2023; Ooki, 2005; Polikowsky et al., 2021; Rautakoski et al., 2012; Raza et al., 2015; Shaw et al., 2021). Despite recent advancements, major gaps in our understanding of the genetic basis for stuttering remain. New genetic approaches have allowed us to explore novel analyses, in particular, estimating modest effects of common variants on polygenic speech/language traits, but conducting robust and replicable genetic studies requires large, well-phenotyped cohorts of subjects (Doust et al., 2022; Eising et al., 2022; Polikowsky et al., 2021; Shaw et al., 2021). Additionally, once genetic variation contributing to stuttering is identified, a key remaining challenge is understanding the biological significance of genetic variation within relevant cell and tissue types, particularly within speech musculature and the brain.

Despite current and past challenges to recruitment and the complexity of characterizing the causes of stuttering, genetic contributions to stuttering have been observed and reported within the literature for nearly 100 years. An early study by Boome and Richardson in 1932 found 71.7% of individuals who stuttered reported a positive family history of stuttering whereas only 14.1% of individuals who did not stutter had a known family history of stuttering (Boome & Richardson, 1932). Another study by Berry (1937) found that stuttering was more common between twins than between siblings and attributed the difference to inheritance.

Modern studies echo this genetic basis for stuttering, with twin studies demonstrating concordance rates ranging from 38% to 62% (Fagnani et al., 2011; van Beijsterveldt et al., 2010). Other studies have continued to show that stuttering is enriched within families (Ambrose et al., 1993; Kraft & Yairi, 2012; Yairi et al., 1996). As one example, in Ambrose et al.'s (1993) study, 45% of participants who stuttered had at least one immediate family member who stuttered, and 71% had at least one extended family member who stuttered, largely mirroring family enrichment reported in earlier studies. Other studies have calculated heritability estimates for stuttering, defined as the proportion of variation in a trait attributed to genetics, with estimates ranging from 0.42 to 0.82 (Dworzynski et al., 2007; Fagnani et al., 2011; Ooki, 2005; Rautakoski et al., 2012; van Beijsterveldt et al., 2010). Together, these estimates of heritability suggest that genetics significantly contribute to stuttering risk (see Table 1).

**Table 1.** Key terms and definitions.

Allele	Different forms of the same gene at a specific location on a chromosome.
Biobank	A repository of biological materials (i.e., DNA) linked with relevant health outcomes.
Deep phenotyping	Ascertainment of detailed measures related to a characteristic/trait of interest such as measures of comorbidities; age at onset; severity; duration; treatment modes/duration; environmental exposure; and behavioral, social, and economic factors.
Gene expression	The process by which information found within a gene is transcribed for downstream biological processes (i.e., creating RNA).
Genetic architecture	A set of genetic features that contribute to an outcome.
Genetic variant	A specific position in the genome that has variable content between individuals. For example, a single-nucleotide polymorphism is a specific type of genetic variant representing a point in the genome in which individuals carry different nucleotides (i.e., A, T, C, or G).
Genome	An individual's complete set of DNA.
Genotype	The specific combination of two nucleotides (one from each chromosome) at a given point in the genome.
Genotyping	The process of measuring genetic variants on both chromosomes at a site of interest. Array-based genotyping measures the genotypes at a set of positions known to vary in the genome, while sequencing comprehensively measures genotypes at all targeted positions (or genome-wide).
Genome-wide association study (GWAS)	A type of genetic analysis that looks at the frequency of common base pair changes and its association with a trait.
Heritability	The proportion of variance in a trait that is explained by genetics.
Linkage disequilibrium (LD)	Nonrandom association of genetic variants, often that are in proximity to each other in the genome. Variants in high LD are inherited together without a recombination event occurring between them more often than independent variants (i.e., those on different chromosomes).
Locus	A specific point in the genome (plural is loci).
Monogenic	Traits or conditions determined by variation in a single gene.
Phenotype	A measured characteristic or trait of interest (e.g., height, smoking status, clinical diagnosis).
Polygenic	Traits or conditions determined by variation in multiple genes, or a combination of genetic and environmental factors.
Polygenic risk score	A measure of genetic risk of a particular trait that is calculated as a combination of effects from multiple variants.
Post-GWAS functional analyses	A variety of approaches employed to provide a biological context to GWAS variants.
Single-nucleotide polymorphism	A specific type of genetic variant in which a single nucleotide (A, G, C, or T) varies within or between individuals.
Trait	Often used interchangeably with phenotype; a measurable characteristic of an individual.
Transcriptome-wide association study	A method used in genetic studies that looks at the association between measured or predicted gene expression and a trait of interest.

## Previous Candidate Genes for Stuttering Identified via Linkage Analysis

Several candidate genes for stuttering have been identified via linkage analyses or family-based genetic analyses. As the name implies, family-based genetic study designs involve ascertaining genetic profiles of affected individuals and their affected and unaffected relatives (i.e., siblings, parents, and larger extended families). These types of studies can pinpoint chromosomal regions shared by affected relatives and likely to harbor a gene or variant that is causal for a trait, and they are well powered in the context of large pedigrees and highly penetrant genetic variation (Teare & Santibañez Koref, 2014). Previous linkage and family-based studies identified significant hits within the genes *GNPTAB*, *GNPTG*, *NAGPA*, *AP4E1*, *DRD2*, *CYP17A1*, and *PPID* (Kang et al., 2010; Kazemi et al., 2018; Lan et al., 2009; Mohammadi et al., 2017; Morgan et al., 2023; Raza et al., 2015). Other studies have sought to understand how these candidate genes impact brain structure and function (Benito-Aragón et al., 2020; Chang et al., 2008; Chow et al., 2020, 2021). For a review of these studies, see Polikowsky et al. (2021) and Chang et al. (2025). Collectively, these findings highlight the potential significance of lysosomal storage genes in brain regions associated with stuttering and point to potential impact on the cortico–basal ganglia–thalamocortical (CBTC) network.

Although research in families and isolated communities has provided insights into potential gene functions associated with stuttering, replication of these findings in studies conducted in other families and the general population has proven elusive and inconsistent (Frigerio Domingues et al., 2019; Gunasekaran et al., 2021; Mohammadi et al., 2017). Specifically, these investigations have not yielded consistent evidence for single-gene, or monogenic, causes of stuttering with broad relevance across populations, thus lending support to the hypothesis that, in most instances, stuttering is a polygenic trait, arising from the combined influence of multiple genetic factors. The hypothesis of these family-based studies is that variants in these genes are causal for stuttering, with a large effect size (high penetrance), yet the failure to replicate these findings suggests that the effect sizes of these variants may have been overestimated. In other words, variants identified in families are rare variants that explain little of the population prevalence of stuttering. Therefore, further research is necessary to fully understand the complexity of genetic factors that influence stuttering risk.

## Genome-Wide Association Studies

In contrast to family-based studies, population-based studies consist of large samples of affected and unaffected individuals who are intended to reflect the genetic landscape of the populations they are drawn from rather than individual families. Genome-wide association studies, or GWASs, examine the influence of common genetic variations across the genome on a specific trait. By statistically testing associations between traits and genetic variants, GWASs can generate hypotheses for further mechanistic studies to understand underlying biological processes. While GWASs have enabled the discovery of thousands of variants for thousands of traits, GWASs require large sample sizes to reach statistical significance because the burden of multiple test corrections is very high and many individual variant effects on disease are small. Indeed, sample sizes for contemporary GWAS publications often number in the tens of thousands to millions. The emergence of large-scale genetic databases linked to electronic health records (EHRs) or survey-based data provides a cost-effective approach for investigating the association between genetic variants and traits of interest at scale. Yet, stuttering is poorly captured in extant biobanks, and therefore, one of the primary challenges in performing GWASs for stuttering is the difficulty of assembling sufficiently large cohorts with both genetic data and stuttering data to enable well-powered statistical analyses. Prior GWASs of stuttering employed a variety of sample collection approaches to address these inherent challenges, which we review below, leading to the discovery of more than 80 novel genetic signals for stuttering, many of which are located in or near genes with neurological functions. However, interpretations of GWAS signals (over 90% of which are noncoding; Altshuler et al., 2008; Hindorff et al., 2009) that rely on physical proximity to a coding gene can often lead to erroneous conclusions about a variant's functional role in a trait (Claussnitzer et al., 2015; Mountjoy et al., 2021; Zhu et al., 2016). To date, multiple approaches have been deployed to map variants identified by GWASs of stuttering to causal genes and interpret their effect, including fine-mapping, colocalization, and genetic correlation analyses. We review these approaches and how they have improved interpretation of findings from GWASs of stuttering. Additionally, we also present new results leveraging a tool not previously implemented in the context of stuttering, transcriptome-wide association study (TWAS).

## Purpose

This article primarily reviews strategies employed by our research team to address challenges in (a) identifying genetic variants associated with stuttering and (b) translating stuttering-associated variants into molecular and cellular mechanisms. An overview of the topics presented in this article was presented as part of an invited talk for the 2024 Research Symposium: Genetics in Communication Sciences and Disorders at the ASHA Convention.

Understanding the underlying genetic architecture of stuttering has long-term potential to contribute to public perceptions of stuttering, providing insights into fundamental mechanisms of human speech and fluency, and illuminating potential targets for precision therapeutics. Specifically, improved understanding of causes of stuttering may replace outdated and inaccurate views of stuttering often held by the general public that contribute to stigma (i.e., that stuttering is a symptom of a personality disorder or results from disadvantageous parenting style). Decades of research have demonstrated that stuttering tends to run in families, and identifying specific genetic elements can further our understanding of answering how stuttering risk is transmitted. Additionally, understanding genetic risk factors may reveal specific subtypes of stuttering with different underlying causes or developmental trajectories. Identification of more precise diagnostic categories could ultimately lead to earlier tailored intervention or the development of novel interventions. Furthermore, genetics may also highlight future research directions and foster collaborations between speech-language pathologists and other specialists, leading to a more comprehensive approach to understanding stuttering. Studies into the genetics of stuttering may lead to critical insights into the etiology of speech conditions and simultaneously highlight mechanisms that drive the production of typical speech, a uniquely human trait.

We also view our work as advocating for research equity: GWASs and large-scale biobank initiatives are common in many areas of biomedical research, and we believe these resources have the ability to advance stuttering research, despite current limitations. We aim to utilize these existing tools to turn the lens on stuttering, a condition with a high degree of genetic heritability but one that has so far been underinvestigated. Overall, the knowledge gained from these studies lays the groundwork for future research, which we hope results in further support for individuals who stutter through more informed and impactful approaches.

## Strategies for Overcoming Sample Acquisition Challenges for Genetic Studies of Stuttering

**Figure 1.**
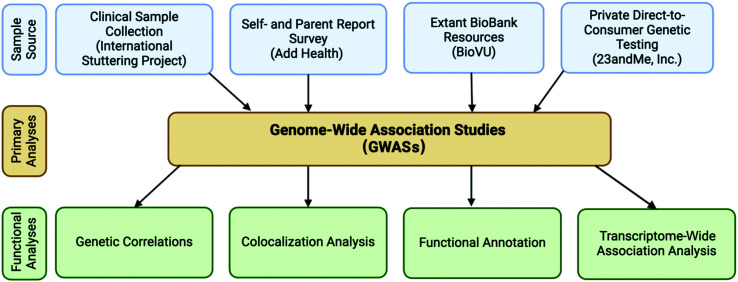
Approaches to understand the genetic etiology of developmental stuttering. GWASs = genome-wide association studies.

Human geneticists who study stuttering have utilized a range of data sources in pursuit of large, well-phenotyped samples for their analyses (see Figure 1). In the following sections, we describe four independent data sets differing in sample sourcing and phenotyping methods. Within each section, we highlight the advantages and limitations of the samples. Then, in the sections following, we describe findings from genetic analyses using these data sets and detail approaches to further explore the impact of genetic variants on stuttering. We believe these various approaches are ultimately complementary in the exploration of the genetics of stuttering.

### The International Stuttering Project—Clinical Sample Collection

Established in 2020, the International Stuttering Project (ISP) is an international collaboration of scientists and speech-language pathologists who specialize in stuttering, many of whom run clinics, practices, and therapy centers for stuttering. The primary goal of the ISP is to expand genetic research by representing diverse global populations of people who stutter. Currently, the ISP consists of 2,071 (male: *n* = 1,455; female: *n* = 616) clinically diagnosed developmental stuttering cases. Participants were recruited from a variety of locations including Curtin Stuttering Treatment Clinics in Perth, Australia; the Linklater Clinic and the Irish Stammering Association, both in Dublin, Ireland; and the National Stuttering Association and Dr. Shelly Jo Kraft's research group at Wayne State University, both in the United States. Each participant enrolled in the ISP is evaluated by a speech-language pathologist highly specialized in developmental stuttering. The majority of these participants are actively receiving speech therapy for stuttering. Participant profiles include case history, persistence and recovery data, clinician-reported severity ratings, and self-report severity ratings. Following assessment, participants provide genetic samples via saliva kits.

In addition to clinically recruited participants, a smaller subsample was recruited via the online science discussion forum on https://www.reddit.com/r/science/. With 34 million subscribers, this forum is dedicated to sharing and discussing new, peer-reviewed scientific research and, in the past, has hosted interviews with prominent research scientists, referred to as “Ask Me Anything” or AMAs. During AMAs, Reddit users can engage with researchers by directly asking questions and sharing their thoughts and concerns about the scientific topic being discussed. Researchers with the ISP held an AMA and provided a link for motivated readers to give informed consent and self-enroll in the study. The initial AMA resulted in 174 people who stutter who self-enrolled in the study. Of these, 127 subsequently had their diagnosis confirmed via phone interview with the study principal investigator and a highly specialized speech-language pathologist and contributed a DNA sample for extraction.

As a result of international recruitment strategies and democratized recruitment from a mass online platform, genotyped participants in the ISP are from diverse genetic ancestries and global regions. A clear advantage of this sample collection approach is the detailed clinical data to pair with the genetic data. Cases are confirmed by speech-language pathologists via assessments and interviews, resulting in high assurance that participants are accurately categorized and profiled. A major drawback of this approach is the time and expense associated with sample collection. The ISP has actively recruited participants for 5 years, and scaling treatment center–based sample collection has proved challenging. Despite a smaller sample size than typical for GWASs, the deep phenotyping of the ISP data has proven vital for validation studies of other sample collection methods.

### The National Longitudinal Study of Adolescent to Adult Health—Self-Report and Parent Report Survey

In addition to clinical recruitment efforts, studies have also utilized survey-based and genetic data from the National Longitudinal Study of Adolescent to Adult Health (Add Health), a data set with self- and parent-reported stuttering. Add Health is an ongoing longitudinal research project examining how social, behavioral, and biological factors impact health and development from early adolescence to adulthood (Harris et al., 2019). Add Health collects a range of data from participants, including demographics, health surveys, in-home data, and biological samples. Within Add Health, stuttering cases were defined as participants who at any point answered “yes” to the survey question: “Do you have a problem with stuttering or stammering?” Controls were defined as participants who answered “no” to the same question at all points. After pre-analysis quality control, this resulted in 785 cases and 7,572 controls (see Material and Methods in Polikowsky et al., 2021), equating to a prevalence of 9.39% in this data set. To improve power for discovery, Add Health stuttering GWAS results were meta-analyzed with the ISP cohort analysis to capture the broader genetic architecture of the stuttering phenotype (e.g., regardless of self-report or clinically defined) in Polikowsky et al. (2021). These data sets were also used to validate the results of the GWASs performed with the large, self-reported 23andMe data set discussed in a later section of this article (see Summary of 23andMe Self-Reported Stuttering GWAS section).

One advantage to this data set is the contemporaneous reporting of stuttering. In contrast, retrospective studies may suffer from less accurate recall from participants, especially those with recovered stuttering. Another advantage is the wealth of longitudinal data and multiple survey data points. Limitations of this data set include the lack of diagnostic confirmation from a speech-language pathologist and a lack of data regarding stuttering severity or impact.

### BioVU—Extant Biobank Resources

Biobanks have rapidly emerged as the de facto approach for efficiently acquiring samples for large epidemiologic and genetic studies. Our prior research innovatively utilized BioVU, the Vanderbilt University Medical Center (VUMC) biobank linked to de-identified EHRs to characterize the phenotypic profile of people who stutter and generate inference models for genetic studies.

As of 2025, the VUMC EHR contains approximately 3.5 million unique patients and de-identified clinical and demographic information including *International Classification of Diseases, Ninth Revision* (*ICD-9*) and *International Classification of Diseases, Tenth Revision* (*ICD-10*) codes, Current Procedural Terminology procedure codes, medications, lab values, and free text from within medical notes. Medical notes detail the reason for the visit; general description of the patient; evaluation of the chief complaint; description of other relevant medical, social, and familial contexts; test results; diagnoses; and follow-up plans, providing rich context for understanding the medical encounter. A subset of patients within the VUMC EHR also has genetic data paired with their EHR via BioVU. Patients receiving blood tests at VUMC can donate their discarded blood samples for DNA extraction, genotyping, and storage in BioVU, which is linked back to their de-identified EHR. By integrating the EHR with genotyped samples, researchers can conduct investigations into genetic variation, disease susceptibility, variability of drug responses, and more. BioVU is one of the largest single-center EHR-linked biobanks in the world with over 300,000 individuals as of 2025.

#### Biobanks and Stuttering

Although biobanks are increasingly used for foundational clinical and genetic research, a significant limitation of many biobanks is the insufficient reporting of communication conditions. For example, attempts to identify developmental stuttering cases via *ICD-9* and *ICD-10* billing codes within the VUMC EHR yielded a prevalence of approximately 0.01%, far below the estimated population prevalence of 1%–3% (Pruett et al., 2021). While billing codes serve as suitable indicators for a wide range of clinical phenotypes, their utility in accurately capturing certain conditions, such as stuttering, is limited. Several factors likely contribute to the underrepresentation of stuttering and similar conditions in EHRs when using basic diagnostic search methods. For example, because most stuttering evaluations occur in schools and/or private clinics, these records often remain separate from traditional medical EHRs. In addition, many speech and language conditions have no billable treatment options such as medical procedures or pharmaceuticals, leading to limited documentation by nurses and physicians. Finally, providers may simply overlook communication conditions during patient encounters when the communication condition is not part of the reason for the encounter. Compounding the challenge, in many large-scale, publicly available biobanks that have collected survey-based data from participants (such as the UK Biobank), information concerning communication conditions is not prospectively collected.

We therefore advocate for better labeling of stuttering within EHRs. This could include increased free-text notation by primary care providers such as writing “patient displays stuttered speech, as per baseline, according to the patient” or by implementing categorical responses such as a check box for any speech, language, or communication differences displayed during the encounter. Both approaches would likely necessitate increased training in communication conditions for physicians but could lead to improvement in both clinical care and the research capabilities of EHRs. We also support prioritization of prospective data collection for stuttering and other communication conditions within extant and future biobanks including through self-report on surveys and questionnaires. Involving experts in the field of communication sciences and disorders as well as affected community members in the early stages of project planning could help prioritize relevant data collection and facilitate interpretation and dissemination while building trust and fostering collaboration.

#### Identifying Stuttering Within BioVU

Even with these challenges, individuals who stutter are routinely seen in medical centers, and records for some of these patients contain sufficient information to identify them as having a stutter. Data regarding stuttering are often found in unstructured free text within medical notes rather than in categorical data such as billing codes (e.g., “Noted that patient displayed stuttered speech during the encounter, per baseline.”). Previous studies by our research team utilized medical notes to identify stuttering within the EHR and to predict underdocumented cases within BioVU (Pruett et al., 2021, 2025; Shaw et al., 2021). First, our team developed a text-mining algorithm of medical notes followed by manual review to identify individuals who stutter. This approach resulted in 1,143 confirmed stuttering cases. From these stuttering cases, we created a machine learning algorithm to predict underdocumented stuttering cases within BioVU based on associated comorbidities. By analyzing diagnostic billing codes that were significantly more common in people who stutter (e.g., neurological disorders, epilepsy, sleep disorders, dermatitis), the model identified patients within BioVU who exhibited a similar pattern of medical diagnoses that we termed the “stuttering-associated phenome.” For a full list of stuttering-associated codes, please see Pruett et al. (2021). We considered these predicted cases as a proxy stuttering phenotype. This approach resulted in 9,239 predicted cases, increasing our sample size for downstream GWAS analyses (Shaw et al., 2021). While we anticipate some phenotypic discrepancy between individuals identified through comorbidity predictions and those identified in clinical settings, subsequent validation studies provide insight into the degree of genetic overlap between the samples.

### 23andMe, Inc.—Private Direct-to-Consumer Genetic Testing

In addition to nonprofit biobanks, private direct-to-consumer genetic testing companies have also contributed mass-scale data for genetic research (Budu-Aggrey et al., 2023; Campos et al., 2023; Choquet et al., 2024; Kmiecik et al., 2024; Niarchou et al., 2021; Pitz et al., 2024). In an effort to increase our sample size for GWASs, we partnered with 23andMe, Inc. through the Research Innovation Collaborations Program. This program invites academic researchers to apply for competitive access to de-identified, aggregated data from the 23andMe Research Cohort to power genetic discoveries (23andMe, 2024). When 23andMe users create an account, they complete a health survey. The survey includes the question: “Have you ever had a stutter or stammer?” For our analyses, cases were participants who answered “yes” (*n* = 99,776) to this question and controls were participants who answered “no” (*n* = 1,023,243), resulting in the largest genetic study of stuttering to date. While this single question is not ideal for phenotyping, many population-based investigations of stuttering rely on retrospective questionnaires or interview-style surveys (Bloodstein & Ratner, 2008). Even with these phenotyping constraints, large-scale sample collection via survey can dramatically increase power, and similar studies have demonstrated robust and reproducible effects for other traits (Hu et al., 2016; Saunders et al., 2022; Watanabe et al., 2022).

A tremendous advantage to the 23andMe data set is the unprecedented sample size of diverse genetic ancestry to harness for genetic discovery of stuttering at little to no direct cost. This represents a major step forward from previous GWASs of stuttering that have suffered from lack of power to observe genetic variants with a modest effect size. Despite the substantial sample size, the wording and retrospective nature of the survey are constraints limiting accuracy for determining stuttering cases and controls. Due to the lack of deeply phenotyped cases within this data set, we have leveraged our other data sets to validate that the stuttering phenotype observed within 23andMe is genetically similar to clinically diagnosed stuttering (Polikowsky et al., 2025). In general, we expect subtle differences between each of these data sets, but overall, the strengths and limitations of each are complementary in genetic discovery for stuttering.

### GWAS Sample Size and Case/Control Selection

GWAS analyses depend on large sample sizes. Without adequately large sample sizes, GWASs can suffer from reduced statistical power to detect variants with small effects and rare variants that have insufficient numbers of observations in cases and controls to estimate robust effects. Sample sizes of published studies have increased dramatically with the development of large-scale biobanks such as the UK Biobank and the All of Us project. For example, early GWAS studies (circa 2000) often featured sample sizes of several hundreds or thousands of cases and controls. Modern GWASs typically feature tens of thousands to hundreds of thousands of cases and controls and may include meta-analyses combining data from multiple studies nearing or even exceeding a million participants (Als et al., 2023; Keaton et al., 2024; Yengo et al., 2022).

In GWASs, accuracy of case–control classification is often balanced by sample size. In contrast to other genetic approaches, one advantage of GWAS is robustness to some degree of case–control misspecification, especially in traits with population prevalence below 20% (Moskvina et al., 2005; Uffelmann et al., 2021). Typically, errors in case–control selection can somewhat diminish power to identify associations but are unlikely to produce spurious associations. In fact, use of population-based controls that are not assessed for case status at all is common in genetic studies of rare traits (Bouaziz et al., 2021; Canela-Xandri et al., 2018; Mitchell et al., 2014; Wojcik et al., 2022). Furthermore, deeply clinically phenotyped cohorts can enhance self-report GWAS results by serving as a validation sample, increasing confidence that the effects observed reflect those of clinically diagnosed cases. Another emerging approach is to infer case/control status from co-occurring conditions, using machine learning approaches called phenotype risk scores (PheRS; Bastarache, 2021; Bastarache et al., 2018; Pruett et al., 2021; Shaw et al., 2021). While the accuracy of PheRS and other case prediction approaches varies by study and by condition, the approaches represent important advances in case selection within EHRs above and beyond the use of traditional billing codes (Bastarache, 2021).

Defining cases and controls through direct clinical assessment of participants and in-depth phenotyping approaches often comes at the cost of smaller sample size, while approaches used to generate large data sets (e.g., self-report or phenotype inference) may result in lower case–control accuracy. For example, Polikowsky et al. (2021), the largest published GWAS of clinically diagnosed stuttering, contained 1,345 cases, whereas Polikowsky et al. (2025) contained 99,776 stuttering cases identified via self-report. In general, self-reported stuttering is highly correlated with speech-language pathologist–judged stuttering (Horton et al., 2024), but additional validation studies would strengthen support for use of self-report within the field.

Overall, a distinct advantage of using extant biobanks and similar resources is the ability to amass substantial samples of genotyped cases and controls at a comparatively low cost. As the field of population genetics has moved toward more collaborative efforts to scale sample collection and analysis, GWAS limitations have been minimized but not yet fully overcome. Studies of height, Type 2 diabetes, cardiovascular disease, and other traits typically involve large consortia for breakthrough findings; the creation of similar consortia for hearing and speech sciences would provide immense opportunities for the field. However, a current limitation is a lack of uniform clinical data for stuttering and other communication conditions within many biobanks. Although we have developed approaches to lessen this issue, as biobanks continue to grow, it is critical to consider the a priori inclusion of communication conditions to ensure that biobank resources can be broadly utilized for the field.

## Overview of Previous Genome-Wide Association Analyses of Stuttering

Leveraging each of these data sets, we summarize our previous GWASs of stuttering in the following sections.

### Summary of ISP GWAS

In Polikowsky et al.'s (2021) study, a stuttering GWAS was performed using data from the clinically ascertained ISP (*n* = 1,345 cases; *n* = 6,759 controls) and the survey-based Add Health (*n* = 785 cases; *n* = 7,572 controls). A significant genome-wide association was found within the gene *SSUH2*, a gene previously implicated in distal myopathy, rippling muscle disease Type 2, and odontogenesis (Xiong et al., 2017). Additionally, 15 more loci reached suggestive significance (*p* < 5 × 10^−5^). While identifying significant and suggestive genetic signals associated with stuttering, the study was limited in power by sample size (*n* = 2,130). This issue is particularly critical considering the hypothesis that many common genetic variants, each with a small effect, contribute to stuttering risk. Despite the limited sample size, the study demonstrated that common genetic variants present in general populations contribute to stuttering risk. Therefore, we sought follow-up studies with larger samples to continue this line of investigation.

### Summary of Phenotypically Predicted BioVU Biobank GWAS

In Shaw et al.'s (2021) study, GWAS of a predicted stuttering phenotype (*n* = 9,239 predicted cases; *n* = 83,503 controls) in BioVU identified a genome-wide significant association near the gene *FAM49A* in participants of primarily European ancestry. *FAM49A* gene expression is elevated in several tissues relevant to stuttering, including the cerebral cortex and basal ganglia, and also in the thyroid and certain immune cells (Uhlén et al., 2015). Additionally, a near-significant association was identified within the gene *ZMAT4* in participants of primarily African ancestry. *ZMAT4* is highly expressed in the central nervous system, including the cerebral cortex, cerebellum, and hippocampus, and is also moderately expressed in the basal ganglia (Uhlén et al., 2015). Results implicating the basal ganglia are especially interesting given that the basal ganglia aid the planning and execution of motor movements, and dysfunction in the CBTC loop has been studied in connection to stuttering (Alm, 2004; Chang & Guenther, 2020; Lu et al., 2010; Nikvarz & Sabouri, 2022; Theys et al., 2013).

Given the unknown clinical specificity of the predicted phenotype, validation of results was performed by creating polygenic risk scores (PRSs) from the predicted stuttering GWAS summary statistics (see Shaw et al., 2021, for full description of methods and results). A PRS summarizes the estimated effect of the genetic variants on an individual's phenotype, typically calculated as a weighted sum of trait-associated variants. In other words, a PRS estimates how likely an individual is to develop a given trait, based entirely on genetics. The PRS developed from the predicted stuttering cases in BioVU significantly predicted case–control status in the clinically ascertained ISP cohort, providing evidence that the genetic architecture identified in the predicted stuttering phenotype is broadly relevant to a clinician-confirmed stuttering phenotype.

### Summary of 23andMe Self-Reported Stuttering GWAS

Polikowsky et al. (2025) investigated self-reported stuttering using data from 23andMe, Inc. The substantial sample size (> 1 million cases and controls) provided by partnership with the 23andMe, Inc. Research Innovation Collaborations Program offered exceptional power to detect stuttering risk alleles with a modest effect size as well as shared and distinct genetic effects across ancestry- and sex-specific groups. Overall, the study identified 24 signals in the primary ancestry- and sex-specific analyses and 63 in secondary meta-analyses (Polikowsky et al., 2025). Of these 87 total signals, 57 were unique loci mapping to 48 distinct genes (Ghoussaini et al., 2021; Mountjoy et al., 2021). To the best of our knowledge, all variants reaching genome-wide significance in this study represent novel discoveries for stuttering.

Many genetic associations identified for stuttering risk were found near genes previously linked to other neurological conditions and traits, supporting a neurological role in stuttering. In particular, *VRK2* has been linked to depression (Turley et al., 2018), schizophrenia (Li & Yue, 2018), and rhythm (Niarchou et al., 2022). The rhythm finding is of particular interest considering studies have shown complex rhythm discrimination is below average in children (Wieland et al., 2015) and adults (Garnett et al., 2023) who stutter and providing external pacing cues such as a metronome can momentarily decrease stuttering (Brady, 1969, 1971; Wingate, 2002). Additionally, *SEMA6D* is involved in pathways related to nervous system development and attention-deficit/hyperactivity disorder (ADHD; Martin et al., 2018), and *SLC39A8* is associated with brain volume measurements (Lam et al., 2019; Zhao et al., 2019). While genome-wide significant findings in Shaw et al. (2021), Polikowsky et al. (2021), and Polikowsky et al. (2025) do not overlap, failure to replicate GWAS findings is common in population-based studies where the expectation is that effect sizes may be small and associated variants can be subject to “winner's curse” (Xiao & Boehnke, 2009). “Winner's curse” is a phenomenon where top GWAS signals are enriched for variants with effects that have been overestimated relative to their true effect size. This renders subsequent studies less likely to find the same variants. Therefore, a key objective of Polikowsky et al. (2025) was validating the similarity of the genetic architecture of 23andMe self-reported stuttering to that of other data sets and diagnostic standards.

As before, PRSs derived from the 23andMe GWAS summary statistics in males and females were applied to the male and female participants in two other independent cohorts: the self- and parent-reported Add Health, and the clinically ascertained ISP, generating an individual-level weighted genetic risk based on the 23andMe summary statistics (Polikowsky et al., 2025). Afterwards, PRS performance was tested within each cohort separately to assess whether genetic risk scores for each individual significantly predicted their actual case/control status. Polikowsky et al. (2025) found that, within Add Health, both male and female 23andMe PRS models significantly predicted case/control status in males and females. In the ISP, a cohort enriched with males and persistent cases of stuttering, the male-derived 23andMe PRS model significantly predicted stuttering cases and controls in both sexes and the female-derived 23andMe PRS model significantly predicted female cases and controls but was not predictive of male case/control status. Full results and methods can be found within Polikowsky et al.'s (2025) study.

The difference in performance between the male and female PRS models in the two independent validation cohorts is notable. Possible nonmutually exclusive explanations include the following: (a) The self-reported female phenotype is less representative of developmental stuttering compared to the self-reported male phenotype, (b) the genetic architecture of developmental stuttering differs between males and females and may be confounded by genetic susceptibility to stuttering recovery or persistence, and (c) genetic variants contributing to stuttering risk may be confounded by other genetic factors modulated by sex (i.e., horizontal pleiotropy). Overall, these analyses provide evidence that stuttering defined via self-report within 23andMe is largely genetically predictive of stuttering in independent self- and parent-reported and clinically ascertained cohorts and suggest that the genetic results obtained from 23andMe are relevant to clinically diagnosed stuttering (see Table 2).

**Table 2.** Summary of population-based stuttering findings.

Study	Phenotype definition	Variants identified	Implicated gene(s)
Polikowsky et al. (2025)	Collaboration with 23andMe survey-based self-reported stuttering (eight ancestry- and sex-specific GWAS and subsequent meta-analyses) Cases: 99,776	57 unique genome-wide significant loci (*p* < 5.00 × 10^−8^)	*VRK2*, *CAMTA1*, *MYO16*, *MMAB*, *CTNND2*, *SEMA6D*, *IRS2*, *COL14A1*, *SLC39A8*, *DCC, SRPK2*, *NMUR2*, *TSHZ2*, *MITF*, *PTPRQ*, *SHISA2*, *CYTH4*, *SORCS1*, *RGCC*, *LRP1B*, *PTBP2*, *GRM5*, *CREB3L4*, *KCTD10*, *UBAP2*, *LYSMD4*, *MAP2K6*, *ARMC2*, *SOX5*, *ZNF567*, *KCNH8*, *ADGRL3*, *TMEM71*, *SGCD*, *MET*, *LRRC69*, *PACRGL*, *CLRN2*, *C16orf95*, *ADCY5*, *CTSC*, *SCN8A*, *AUTS2*, *PLPPR1*, *POLI*, *BMPR1B*, *ZFP64*, *CBLN4*
Polikowsky et al. (2021)	Meta-analysis of: Clinically ascertained stuttering (ISP; cases: 1,345) Self-reported stuttering (Add Health; cases: 785) Total meta-analyzed case *N*: 2,130	1 genome-wide significant loci 15 loci reaching suggestive significance (*p* < 5.00 × 10^−6^)	*SSUH2*
Shaw et al. (2021)	Individuals predicted to stutter in electronic health records Cases: 9,221	1 genome-wide significant loci 11 loci reaching suggestive significance (*p* < 5.00 × 10^−6^)	*CYRIA* (also referred to as *FAM49A*)

*Note.* GWAS = genome-wide association study; ISP = International Stuttering Project; Add Health = National Longitudinal Study of Adolescent to Adult Health.

### Limitations of GWASs

While GWASs have been a powerful tool for identifying genetic variants associated with a wide range of complex traits, conditions, and diseases, as with any single tool, there are limitations to the approach. First, as the name suggests, GWASs identify associations between genetic variants and traits and conditions but do not establish causal relationships. In addition, many variants identified in GWASs occur in areas of DNA that do not encode a protein (noncoding regions), making it challenging to understand their functional impact on downstream mechanisms. Linking genetic variants identified in GWASs to causal genes and regulatory mechanisms requires integration with other functional genomics data and experimental validation, approaches we discuss and advocate for within this article.

Second, single-nucleotide polymorphism (SNP)-based heritability estimated from additive effects of measured genetic variants typically explains only a small portion of the overall family-based heritability of a trait, which are often derived from twin studies, an issue commonly known as “missing heritability.” There are many explanations for this missing heritability (Sandoval-Motta et al., 2017). For example, estimates can be biased in the presence of admixture and population substructure, GWASs may lack power to detect rare variants that contribute to a trait as well as variants with small effects, and structural variants such as deletions, duplications, and inversions are often poorly interrogated in GWASs. Furthermore, epistatic (e.g., gene-by-gene) interactions are not detected in standard GWASs. Finally, the effect of a variant may depend on environmental factors, which may not be modeled in GWASs.

Third, population stratification, or differences in genetic variant frequency due to differences in genetic ancestry or relatedness among study participants, can lead to spurious associations if not carefully controlled in the GWAS. Most GWASs have been conducted in populations of predominantly European genetic ancestry, limiting the generalizability of findings across populations and potentially missing important genetic effects in non-European people. Efforts to diversify GWASs at scale are underway for many traits, and the recent article by Polikowsky et al. (2025) is the first to explore GWASs of stuttering in diverse populations.

## Functional Analyses

**Figure 2.**
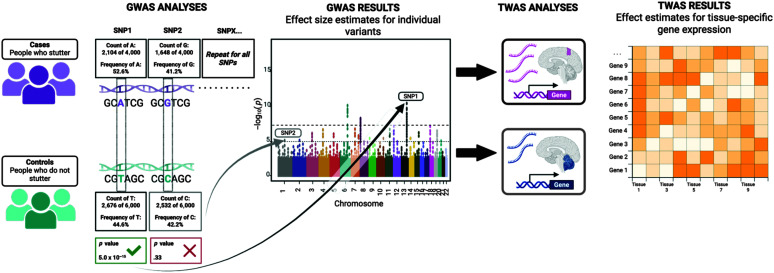
Overview of genome-wide association study (GWAS) and its association with transcriptome-wide association study (TWAS). GWASs examine the frequency of common base pair changes (i.e., single-nucleotide polymorphisms [SNPs]) and their association with a trait. “GWAS ANALYSES” results include effect size estimates for individual variants and are often displayed on a Manhattan plot. Here, the “GWAS RESULTS” *x*-axis represents genomic position by chromosome, and the *y*-axis represents the statistical significance of the association between a variant and stuttering. “TWAS ANALYSES” examine the association between measured or genetically predicted gene expression and a trait of interest in specific tissues. Here, “TWAS RESULTS” include predicted levels of tissue-specific gene expression. The heat map has tissues along the *x*-axis with genes on the *y*-axis, with color saturation representing predicted levels of gene expression.

Identifying genetic associations via GWASs is the first step in discovery, but follow-up studies are crucial to translating associated genetic variants into biological mechanisms of action. As described in the previous sections, population-based GWASs have identified more than 50 variants associated with stuttering in independent samples. Progressing from statistical associations of stuttering with genetic variants to understanding the functional genetic mechanisms that influence speech is a challenging process (Schaid et al., 2018). Inferring biological context can be difficult in GWASs, as most genetic signals occur in noncoding parts of the genome (Altshuler et al., 2008; Hindorff et al., 2009), complicating interpretation of how genetic variation directly contributes to traits. Consequently, various methods have been developed to help interpret GWAS signals, often focused on gene expression, highlighting the importance of gene expression in trait variation (Nicolae et al., 2010). See Figure 2 for an overview of GWAS and post-GWAS analyses. In the following sections, we describe four post-GWAS analyses (genetic correlation, fine-mapping by colocalization, functional annotation, and TWASs) and present novel results from TWAS analyses of the ISP cohort.

### Genetic Correlation Analyses

Genetic correlation analysis is a post-GWAS analysis approach that quantifies the extent to which two or more traits share underlying genetic influences (Bulik-Sullivan et al., 2015). A high genetic correlation suggests that many of the same genetic variants contribute to both traits with similar magnitudes of effect, illuminating biological commonalities between traits even when relationships are not phenotypically apparent. A significant genetic correlation suggests that some of the same genetic variants influence the susceptibility or severity of both conditions and points toward shared underlying biological pathways, genes, or molecular mechanisms. Together, these results can further help us understand the type of traits that may be associated with stuttering on a genetic level. While genetic correlation itself does not establish causality, it can inform Mendelian randomization (MR) studies, which use genetic variants as instrumental variables to statistically infer causal relationships between traits. A strong genetic correlation might suggest potential causal links worth investigating with functional studies or via statistical causal inference approaches such as MR. In essence, a significant genetic correlation warrants further investigation to understand the nature of the shared genetic influences at the phenotypic level.

Mounting evidence suggests stuttering is associated with a variety of comorbidities (Blood et al., 2003; Pruett et al., 2021). Following our 23andMe GWAS, genetic correlation analyses were conducted using the male and female 23andMe summary statistics and publicly available GWAS summary statistics for 18 traits previously associated with stuttering in the literature (Polikowsky et al., 2025). For females who stutter, genetic correlation analysis yielded significant positive correlations with depression, hearing loss, asthma, daytime sleepiness, ADHD, body mass index, and autism as well as negative genetic correlations with alcohol consumption, walking pace, and musical beat synchronization. For males who stutter, genetic correlation analysis yielded significant positive correlations with depression and autism as well as negative genetic correlations with musical beat synchronization. On the whole, the direction of these genetic correlations is consistent with previous literature findings.

Broadly, this analysis provides evidence for a genetic explanation for the co-occurrence of traits, which may ultimately be useful in understanding shared mechanisms and the development of novel therapy approaches. Notably, these analyses were restricted to traits with publicly available sex-specific summary data with sample sizes of *n* > 1,000, so many other traits of interest from the literature, such as motor and language traits, were not available and were not tested. These analyses focused on comorbidities present in the literature; genetic correlations with previously unexamined traits may prove informative in the future.

### Fine-Mapping by Colocalization Analysis

The structure of the human genome causes variants in proximity to one another to exhibit linkage disequilibrium, and as a result, genome-wide significant loci often range in width from many kilobases to several megabases in size. Fine-mapping is a process that can identify and help prioritize and quantify the strength of functional genetic variants within a significant locus for additional study (Schaid et al., 2018). Biological context can be further probed by inferring whether associated genetic variants influence gene expression within specific tissues. This type of analysis, called colocalization analysis (Hukku et al., 2022), can probabilistically determine if variants with effects on stuttering also show effects on gene expression (called expression quantitative trait loci or eQTLs) in human tissues. A commonly used external data set of eQTLs comes from the Genotype-Tissue Expression project (The GTEx Consortium, 2015), which is a publicly available tissue and gene expression resource. GTEx contains RNA sequencing data for 54 nondiseased human tissue sites. While relatively comprehensive, some key tissues related to stuttering still lack publicly available expression data. New resources will provide additional developmental insight, which may be especially important for traits such as stuttering. For example, the Developmental GTEx Project aims to study genetic effects on gene expression from infancy through puberty. Recruitment for the project began in 2023, and data analysis is underway.

Colocalization analysis was performed on the ISP GWAS (Polikowsky et al., 2021) and the 23andMe GWAS (Polikowsky et al., 2025). For the ISP GWAS, colocalization identified three regions that have a probability of having shared genetic signals between the variant and eQTL data. The variants identified were rs10779884, which affects gene expression of *FBLN* in skeletal muscle, esophagus mucosa, and hypothalamus brain tissues, and rs140321250, which affects gene expression of *INPP4A* in the esophagus mucosa tissue. The *FBLN7* gene encodes a protein that influences molecules within the cellular matrix of developing tooth structures and is crucial for the development of odontoblasts, the cells responsible for producing dentin, the hard tissue beneath the tooth enamel (UniProt Consortium, 2021). The gene *INPP4A* encodes an enzyme requiring magnesium ions to break down a specific part of the Vitamin B8 molecule (Joseph et al., 1998).

This analysis was also performed in the 23andMe GWAS. Colocalization analysis identified rs12314392, which affects gene expression of *MMAB* (involved in Vitamin B12 catalyzation) and *MVK* (associated with cholesterol biosynthesis and metabolism of steroids) in the following tissues: the gastroesophageal esophagus junction, left ventricle of the heart, transverse colon, whole blood, atrial appendage of the heart, visceral omentum adipose tissue, tibial artery, sigmoid colon, cultured fibroblast cells, esophagus mucosa, and aortic artery. Overall, these results suggest that these variants may be affecting gene expression speech-relevant tissues and neural correlates. A limitation of these analyses is that they are computationally probabilistic in nature. Therefore, these results do not directly reveal the mechanism of action for a particular variant but can be used to predict potential downstream mechanisms.

### Functional Annotation

To interpret function, genomic tools incorporate information from other functional databases and data sources (i.e., chromatin interactions, in silico functional predictions, distance between gene and transcription start cite) to prioritize biological meaning (Ghoussaini et al., 2021; Mountjoy et al., 2021; Wang et al., 2010; Watanabe et al., 2022). Functional annotation can map genetic variants to genes, regulatory elements, and/or functional information to better understand potential biological impact. Within the stuttering GWAS performed using clinically ascertained stuttering data, the genome-wide significant variant (rs113284510) was able to be mapped to *SSUH2* (Polikowsky et al., 2021). Additionally, the GWAS of a predicted stuttering phenotype was able to map the genome-wide significant variant (rs12613255) to *FAM49A* (Shaw et al., 2021). Last, in the 23andMe self-reported stuttering GWAS, the 57 distinct genomic signals were able to be mapped to 48 unique genes (Polikowsky et al., 2025). By using annotation, we can take variant-level statistical associations and further understand and assess biological context in the genome.

However, functional annotations are limited by the accuracy and completeness of existing databases, with many databases biased toward well-studied genes and pathways. Additionally, interpreting the distance between a gene and regulatory elements is complex and context specific, so linear distance is not always the best proxy for function.

### TWASs

While GWAS maps individual genetic variant effects on a trait, TWASs model the effects of gene expression on a trait. TWASs are useful because, unlike single variants, which may have unknown function, the results of a TWAS are naturally biologically interpretable. Specifically, TWASs of genetically regulated gene expression (GReX) sum individual variant effects for a given gene weighted by their effect on that gene's expression, resulting in tissue-specific predicted gene expression. TWASs can be performed using individual-level genetic data or GWAS results on a trait (Gamazon et al., 2015; Zhou et al., 2020). When used with expression prediction models constructed in GTEx, TWASs can identify tissue-specific gene expression associated with a given trait in 54 different human tissues, computationally prioritizing genes and tissues for functional follow-up in model systems. Because TWASs combine effects of SNPs that regulate a gene's expression level in a given tissue, sometimes novel genes (i.e., not found in GWASs) emerge from TWAS analyses. Another advantage of TWASs is that they are often better powered than GWASs because fewer statistical tests are performed. The ability to find new genes, reduced multiple testing correction, and orientation around tissue-specific gene function are features that make TWASs attractive for investigators. While many TWASs have been successfully deployed to interpret associated genetic variants for a variety of traits (Chen et al., 2021; Gamazon et al., 2019; Tang et al., 2023; Yao et al., 2021), the studies we present below are the first use of TWASs to illuminate mechanisms influencing stuttering risk.

#### Novel TWAS Results From ISP

**Figure 3.**
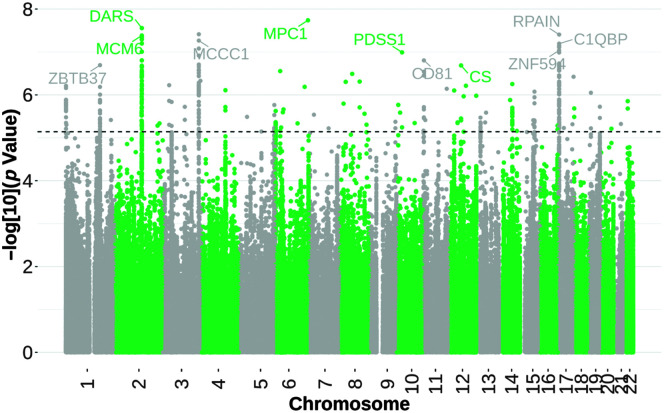
International Stuttering Project transcriptome-wide association study (TWAS). Transcriptome-wide association results for the clinically ascertained stuttering cohort, the International Stuttering Project. TWAS analyses were run on *n* = 1,345 cases and 6,759 matched controls, controlling for the first six principal components. The *x*-axis represents chromosome and base pair coordinates in the human genome, and the *y*-axis represents observed prediction *p* value for each analysis, with alternating colors showing the distinction of chromosomes.

Here, we present new analyses to better understand the functional context of our clinically diagnosed stuttering cases from the ISP. We conducted a follow-up TWAS to predict tissue-specific GReX. The TWAS was performed on individual-level genetic data (*n* = 1,345 cases; *n* = 6,759 matched controls) controlling for the first six principal components (computed using PLINK [Version 19]; Purcell et al., 2007) using the trained tissue models, which leveraged cross-tissue gene-expression similarities (Zhou et al., 2020). We found that the GReX of 81 unique genes was associated with clinically ascertained stuttering across 49 available tissues within the GTEx database at the time of analysis, after using a Bonferroni correction within tissues and a study-wide false discovery rate correction, adjusted *p* < .05 (see Figure 3). Top genes consisted of *MCCC1*, which is responsible for making an alpha subunit of an enzyme found in the mitochondria that breaks down leucine within cells found in 18 different tissues including pituitary, heart, and esophagus tissues, and *RPAIN*, which is involved in enabling metal ion–binding activity found in 18 different tissues including the brain, adipose, and esophagus tissues (Gallardo et al., 2001; Online Mendelian Inheritance in Man).

The gene *MCCC1* (GWAS Catalog: GCST90308590) has been previously associated with Parkinson's disease (Ibanez et al., 2017). Recent evidence points to connections between Parkinson's disease and acquired neurogenic stuttering (Hartelius, 2014; Tsuboi et al., 2019; Tykalová et al., 2015) or stuttering that begins in adulthood due to a neurological injury or disease (Gooch et al., 2023). While the mechanisms leading to acquired neurogenic stuttering are poorly understood, basal ganglia circuits are thought to play a role in stuttering following traumatic brain injury and drug use (Alm, 2004; Pruett et al., 2025; Theys et al., 2013). Furthermore, *RPAIN* (GWAS Catalog: GCST90128471) was found to be previously associated with schizophrenia, which shares certain traits with stuttering such as sleeping difficulties as well as slower and tense muscle movements (Trubetskoy et al., 2022). In addition to these associations in brain regions, these results complement previous findings suggesting that stuttering may be neurologically influenced.

A limitation of this analysis is that many transcriptome reference models used for TWASs are generated and trained using data from individuals of European ancestry and may not adequately represent genetic effects in other ancestry groups, which may be under different environmental and genetic pressures. This has driven calls to increase diversity in TWAS reference data. Furthermore, the latest sample sizes for transcriptomic data in these tissues vary greatly, with the highest number of samples found in skeletal muscle (*n* = 818), while other samples presumed to be more relevant to stuttering (i.e., samples from 13 different brain regions) vary from *n* = 181 to *n* = 289 (GTEx V10). Smaller sample sizes for brain areas may affect the power to predict associations in these tissues. Lastly, we would like to highlight that results from these analyses predict the GReX with a trait of interest within a specific tissue. These results do not establish causation and emphasize the importance of integrating computational genetics work with laboratory-based functional studies.

## Discussion

Twin studies, family-based and linkage analyses, and GWASs continue to demonstrate a genetic basis for stuttering risk. Stuttering research, including our own, has utilized a variety of data sources in pursuit of overcoming previous limitations in robust and reproducible identification of genetic variation associated with stuttering. Each of these data sets has advantages and limitations but together work in synthesis to interrogate and interpret the genetic architecture of stuttering.

While the genetic material in all cells is the same (somatic variation aside), not all genes are expressed in all tissues; it may seem counterintuitive that many genes identified in studies of stuttering do not have direct involvement in known neurological speech-motor pathways. Because GWASs are hypothesis-free studies, it is common to identify variants in or near genes that do not have a previously understood connection to the trait under study. As a well-known example, disruptions in the gene *FOXP2* have been shown to be causal for childhood apraxia of speech within certain families, transmitted from affected parents to roughly half of the children in a monogenic autosomal-dominant manner (Lai et al., 2001). Despite the clear genetic cause, the downstream effects of how variants of *FOXP2* lead to childhood apraxia of speech are still under investigation. *FOXP2* encodes a transcription factor (Vernes et al., 2007), which can increase or decrease the activity of other genes. Importantly, *FOXP2* expression is not exclusive to speech-language areas of the brain or even to the central nervous system: It is active in many other organs and tissues in the body (Schroeder & Myers, 2008) and is even present in many other nonhuman organisms (Fisher & Marcus, 2006). Thus, even when a single causal gene is identified, it can be very difficult to pinpoint how the gene directly influences the onset of the condition. The complexity of unraveling biological mechanisms is compounded for polygenic conditions such as stuttering, where many genes of small or modest effect contribute to the trait. This suggests that stuttering might involve biological pathways or systems in the body that are not yet fully understood. For this reason, exploratory analyses such as GWASs represent a step toward new mechanistic understanding and should be complemented with additional computational and functional studies.

Functional analyses such as TWASs probe the impact of variants within tissues relevant to the trait, taking us closer to understanding possible mechanistic pathways in the development of stuttering. However, understanding how specific regions of the genome contribute to traits such as stuttering remains complex; the genetic variations involved likely have subtle molecular and cellular effects, and human speech is a multidimensional endeavor.

Future research will require investigations from computational, cellular, and animal model approaches as no single approach is likely sufficient to draw causal conclusions. Often, genetic variants identified via GWASs are associated with other phenotypes (Watanabe et al., 2019) and are strongly correlated with other neighboring genetic variants due to linkage disequilibrium (Slatkin, 2008). As a result, establishing the causal variants and their mechanism of causal effect is challenging (Uffelmann & Posthuma, 2021). Cellular models can provide a way to study the functional impact of genetic variants identified through GWASs in a controlled and experimentally tractable system. These approaches have provided additional context for GWAS findings across various neurodevelopmental and communication conditions. For example, induced pluripotent stem cells (iPSCs) can be derived from somatic cells, reprogrammed to a pluripotent state, and differentiated into various cell types such as neurons and glial cells (H. Liu & Zhang, 2011). These iPSC-derived neurons have been used to investigate autism, and broadly, these studies revealed that numerous autism risk variants initially identified via GWASs lead to alterations in neurogenesis, synaptic function, and gene expression (Cheffer et al., 2020; Deshpande et al., 2017; Griesi-Oliveira et al., 2021; X. Liu et al., 2017). Cellular models have also contributed to our understanding of schizophrenia, a neurodevelopmental disorder, by investigating the functional roles of GWAS-linked genes in transcriptional regulation and synaptic function (Brennand et al., 2011; Craddock et al., 2007; Forrest et al., 2017; Millan et al., 2016; Seshadri et al., 2017; Wen et al., 2014). These methods hold promise for better characterizing the role of genetic variants identified in GWASs of stuttering.

Animal models provide another level of experimental validation for GWAS findings, adding organismal-level complexity to functional interpretations. Mouse models engineered to carry human stuttering-associated gene mutations, such as those in *GNPTAB*, have been found to exhibit altered patterns of ultrasonic vocalizations, including longer pauses and increased syllable repetitions, which resemble aspects of human stuttering (Han et al., 2019). Songbird models, particularly zebra finches, have also been used to study song irregularities that share similarities with part-word repetitions observed in human stuttering, suggesting potential parallels in the neural mechanisms underlying these vocal behaviors that could be used to further explore genetic loci influencing stuttering (Chakraborty et al., 2017).

These examples underscore how both cellular and animal models can help bridge the gap between genetic associations and biological function. However, despite their utility to study vocalizations, these animal models do not fully replicate the complexity of human speech, which involves sophisticated linguistic processing and cognitive control, as well as the emotional and social aspects associated with stuttering in humans. Each approach has unique advantages and limitations, but by combining computational human genetic studies with the experimental capabilities of cellular and animal models, researchers can achieve a more complete understanding of the neurobiological basis of stuttering.

Human communication is complex and multifaceted; it is crucial to understand that genetic research alone is unlikely to provide a complete and comprehensive explanation of stuttering. Investigating the genetic architecture of stuttering offers a mechanistic framework that unites previous research, which revealed stuttering to be highly heritable, with research demonstrating neurophysiological differences in people who stutter. Genetic studies may advance early detection, therapeutic approaches, and accommodations to improve the quality of life for people who stutter.

However, genetic factors are rarely, if ever, deterministic. They do not imply a lack of capacity for change or diminish the unique and varied experiences of individuals who stutter. Consistent with the evolving neurodiversity movement, our investigations into genetic factors involved in the development of stuttering do not intend to suggest that stuttering is a deficit but rather a part of human diversity worthy of study. The path forward should include not only the voices of the scientific community but also those of adults and children who stutter, their families and caregivers, and the intersection of these communities: researchers who stutter. For our future work, we hope to continue to work in partnership with the stuttering community in reducing stigma and developing the health care research agenda.

## Conclusions

This review highlights the challenge of acquiring samples for stuttering, the challenge of interpreting functional context following GWASs, and the critical need for improved data collection efforts for speech, language, and communication traits. One reason stuttering research lags behind other common complex traits is the difficulty in amassing large data sets with detailed information on individuals who stutter. Therefore, despite nearly 3 decades of GWAS research on complex traits, applying these approaches to study stuttering is nascent. Given the prevalence and potential impact of stuttering, this lack of robust genetic and clinical data for stuttering represents a major gap. As the field of genetics moves to increasingly sophisticated multi-omic and single-cell functional genetic approaches, it will be critical for stuttering research to keep pace with these advancements in large-scale research efforts to ensure scientific representation. We advocate for better labeling of stuttering within EHRs and prioritization of prospective data collection for stuttering and other communication conditions within extant and future biobanks.

## Data Availability Statement

The original data presented for transcriptome-wide association studies are available in Supplemental Material S1. For the availability of other data, please refer to the original referenced manuscripts.

## Supplementary Material

10.1044/2025_JSLHR-25-00093SMS1 Supplemental Material S1 Transcriptome-wide association results for the International Stuttering Project.

## Notes

**Publisher Note:** This article is part of the Forum: Research Symposium on Genetics in Communication Sciences and Disorders.
