# Supplementary material for: Challenges and Opportunities in Characterizing the Genetics of Stuttering: From Sample Acquisition to Functional Interpretation of the Genome
Source: J Speech Lang Hear Res. 2025 Oct 17;68(11):5137–57. doi: 10.1044/2025_JSLHR-25-00093 (PMC12614926; doi:10.1044/2025_JSLHR-25-00093)
Supplement: Supplemental Material S1 [file JSLHR-68-5137-s001.pdf]

Supplemental material, Pruett et al., “Challenges and Opportunities in Characterizing the Genetics of Stuttering: From Sample Acquisition to Functional Interpretation of the Genome,” *JSLHR*, https://doi.org/10.1044/2025_JSLHR-25-00093

Supplemental Material S1. Transcriptome-wide association results for the International Stuttering Project.

Ensembl Gene ID	Gene name	effect	se	zscore	pvalue	n_samples	status	tissue	bonferroni	FDR	Tissue_Combined
ENSG00000255289	None	-0.2576814	0.04380823	-5.8820322	4.22E-09	8011	NA	Testis	7.04E-05	0.00223978	Testis
ENSG00000060762	MPC1	-0.216908	0.03850621	-5.6330648	1.83E-08	8011	NA	Esophagus_Muscularis	0.00024672	0.00274007	Esophagus_Muscularis
ENSG00000115866	DARS1	0.11979296	0.02154408	5.56036532	2.78E-08	8011	NA	Ovary	0.000243	0.00274007	Ovary; Heart_Left_Ventricle; Muscle_Skeletal; Colon_Transverse; Vagina; Uterus
ENSG00000243368	MCCC1-AS1	0.15634236	0.0284131	5.50247406	3.86E-08	8011	NA	Colon_Transverse	0.00049416	0.00274007	Colon_Transverse; Artery_Tibial; Artery_Aorta; Minor_Salivary_Gland; Esophagus_Gastroesophageal_Junction; Pancreas; Artery_Coronary; Skin_sun_exposed_lower_leg; Colon_Sigmoid; Breast_Mammary_tissue; Thyroid; Esophagus_Muscularis; Nerve_Tibial; Ovary; Testis; Adrenal_Gland; Adipose_Subcutaneous; Prostate
ENSG00000129197	RPAIN	-0.0630654	0.01146752	-5.4994796	3.93E-08	8011	NA	Adipose_Visceral	0.00051684	0.00274007	Adipose_Visceral; Testis; Breast_Mammary_tissue; Brain_Anterior_cingulate_cortex; Heart_Atrial_Appendage; Cells_Cultured_fibroblasts; Adipose_Subcutaneous; Esophagus_Mucosa; Esophagus_Gastroesophageal_Junction; Colon_Transverse; Esophagus_Muscularis; Stomach; Lung; Artery_Aorta; Skin_not_sun_exposed_suprapubic; Brain_Cortex; Brain_Hypothalamus; Pancreas
ENSG00000076003	MCM6	0.11974435	0.02181017	5.4902991	4.14E-08	8011	NA	Colon_Transverse	0.00052932	0.00274007	Colon_Transverse; Thyroid; Whole_Blood; Lung; Spleen; Artery_Coronary; Adipose_Subcutaneous; Esophagus_Muscularis; Esophagus_Gastroesophageal_Junction; Breast_Mammary_tissue; Artery_Tibial; Esophagus_Mucosa; Prostate; Heart_Atrial_Appendage; Adipose_Visceral; Artery_Aorta; Cells_Cultured_fibroblasts; Heart_Left_Ventricle; Uterus; Ovary; Adrenal_Gland; Small_Intestine_Terminal_Ileum
ENSG00000226476	LINC01748	0.15967493	0.02914867	5.47794837	4.43E-08	8011	NA	Esophagus_Mucosa	0.00060307	0.00274007	Esophagus_Mucosa
ENSG00000078070	MCCC1	-0.0909143	0.0167065	-5.4418523	5.43E-08	8011	NA	Adipose_Subcutaneous	0.00074597	0.00274007	Adipose_Subcutaneous; Adipose_Visceral; Artery_Aorta; Artery_Tibial; Ovary; Esophagus_Muscularis; Thyroid; Heart_Left_Ventricle; Nerve_Tibial; Colon_Sigmoid; Liver; Testis; Artery_Coronary; Breast_Mammary_tissue; Stomach; Lung; Prostate; Colon_Transverse; Skin_not_sun_exposed_suprapubic; Skin_sun_exposed_lower_leg; Esophagus_Gastroesophageal_Junction; Kidney_Cortex; Cells_EBV_transformed_lymphocytes; Heart_Atrial_Appendage; Adrenal_Gland; Small_Intestine_Terminal_Ileum
ENSG00000108561	C1QBP	0.21502548	0.03972715	5.4125579	6.39E-08	8011	NA	Lung	0.00086566	0.00274007	Lung; Adipose_Visceral; Liver; Prostate; Heart_Left_Ventricle; Skin_not_sun_exposed_suprapubic; Adipose_Subcutaneous; Thyroid; Breast_Mammary_tissue; Whole_Blood; Nerve_Tibial; Heart_Atrial_Appendage; Artery_Tibial; Esophagus_Muscularis; Esophagus_Gastroesophageal_Junction; Skin_sun_exposed_lower_leg; Testis; Brain_Cerebellum; Brain_Cortex; Brain_Cerebellar_Hemisphere; Brain_Hypothalamus
ENSG00000180626	ZNF594	-0.1190249	0.02225952	-5.347148	9.18E-08	8011	NA	Brain_Cortex	0.00096067	0.00274007	Brain_Cortex; Brain_Anterior_cingulate_cortex; Brain_Putamen_basal_ganglia; Brain_Hypothalamus
ENSG00000144224	UBXN4	0.08756198	0.01641475	5.33434841	9.85E-08	8011	NA	Pancreas	0.00107729	0.00274007	Pancreas
ENSG00000263272	None	-0.0783644	0.01469713	-5.3319514	9.98E-08	8011	NA	Adipose_Visceral	0.00131385	0.00274007	Adipose_Visceral; Cells_Cultured_fibroblasts; Esophagus_Muscularis; Esophagus_Gastroesophageal_Junction; Brain_Amygdala; Adipose_Subcutaneous; Heart_Left_Ventricle; Brain_Hypothalamus; Esophagus_Mucosa; Brain_Cerebellar_Hemisphere; Brain_Cortex; Brain_Putamen_basal_ganglia; Ovary; Minor_Salivary_Gland
ENSG00000148459	PDSS1	0.0869792	0.01632674	5.32740864	1.02E-07	8011	NA	Liver	0.00084445	0.00274007	Liver
ENSG00000110651	CD81	0.51107707	0.09736763	5.24894219	1.57E-07	8011	NA	Brain_Frontal_Cortex	0.00145023	0.00323761	Brain_Frontal_Cortex; Brain_Anterior_cingulate_cortex; Brain_Hippocampus; Heart_Left_Ventricle; Brain_Putamen_basal_ganglia; Small_Intestine_Terminal_Ileum; Brain_Cerebellar_Hemisphere
ENSG00000185278	ZBTB37	-0.3318491	0.06380086	-5.2013266	2.03E-07	8011	NA	Whole_Blood	0.00209482	0.00323761	Whole_Blood; Esophagus_Mucosa
ENSG00000062485	CS	0.09839026	0.01892711	5.19837605	2.06E-07	8011	NA	Esophagus_Mucosa	0.00280227	0.00323761	Esophagus_Mucosa
ENSG00000180596	H2BC4	0.25582101	0.04975378	5.14174009	2.79E-07	8011	NA	Heart_Left_Ventricle	0.00316294	0.00323761	Heart_Left_Ventricle
ENSG00000171316	CHD7	-0.1378709	0.026962	-5.1135252	3.24E-07	8011	NA	Thyroid	0.00474743	0.00323761	Thyroid
ENSG00000173818	ENDOV	-0.0620623	0.01220896	-5.0833398	3.79E-07	8011	NA	Artery_Coronary	0.00386191	0.00323761	Artery_Coronary
ENSG00000108559	NUP88	-0.0292532	0.00575734	-5.0810269	3.84E-07	8011	NA	Testis	0.00641353	0.00323761	Testis; Brain_Cerebellum; Brain_Cerebellar_Hemisphere; Muscle_Skeletal
ENSG00000121988	ZRANB3	0.17656025	0.03477096	5.07780752	3.90E-07	8011	NA	Brain_Hippocampus	0.00317101	0.00324131	Brain_Hippocampus; Brain_Caudate_basal_ganglia; Artery_Tibial; Brain_Cerebellum; Brain_Cortex; Whole_Blood
ENSG00000152492	CCDC50	0.07591033	0.01505517	5.0421419	4.70E-07	8011	NA	Vagina	0.00344178	0.00378732	Vagina
ENSG00000156486	KCNS2	-0.1761701	0.034995	-5.0341502	4.90E-07	8011	NA	Esophagus_Muscularis	0.00661106	0.00382913	Esophagus_Muscularis; Colon_Sigmoid
ENSG00000104299	INTS9	0.19953362	0.03965693	5.03149387	4.97E-07	8011	NA	Spleen	0.0057493	0.00382913	Spleen
ENSG00000029725	RABEP1	0.15091298	0.03006894	5.01889925	5.31E-07	8011	NA	Brain_Frontal_Cortex	0.0049069	0.00391768	Brain_Frontal_Cortex; Brain_Cortex; Brain_Nucleus_accumbens_basal_ganglia
ENSG00000072415	PALS1	-0.1641251	0.03276832	-5.0086513	5.60E-07	8011	NA	Colon_Sigmoid	0.00690844	0.00397086	Colon_Sigmoid; Adipose_Visceral; Artery_Tibial
ENSG00000153551	CMTM7	0.14309284	0.02863695	4.99679004	5.95E-07	8011	NA	Skin_sun_exposed_lower_le g	0.00834326	0.00397086	Skin_sun_exposed_lower_leg; Pancreas; Minor_Salivary_Gland
ENSG00000111052	LIN7A	-0.1371753	0.02748453	-4.9910019	6.13E-07	8011	NA	Testis	0.0102469	0.00397086	Testis
ENSG00000169962	TAS1R3	0.38019481	0.07619711	4.98962255	6.18E-07	8011	NA	Testis	0.0103201	0.00397086	Testis; Artery_Tibial; Thyroid; Cells_EBV_transformed_lymphocytes; Artery_Aorta; Brain_Cerebellum; Whole_Blood
ENSG00000203722	RAET1G	-0.2665517	0.05352787	-4.9796814	6.50E-07	8011	NA	Cells_Cultured_fibroblasts	0.0083004	0.00397086	Cells_Cultured_fibroblasts
ENSG00000183831	ANKRD45	0.13306519	0.02672411	4.97921808	6.52E-07	8011	NA	Adipose_Subcutaneous	0.00895569	0.00397086	Adipose_Subcutaneous; Nerve_Tibial; Cells_Cultured_fibroblasts; Skin_sun_exposed_lower_leg; Adipose_Visceral; Adrenal_Gland
ENSG00000149573	MPZL2	0.16635184	0.03354163	4.95956345	7.21E-07	8011	NA	Brain_Frontal_Cortex	0.00666464	0.00408948	Brain_Frontal_Cortex
ENSG00000172399	MYOZ2	-0.0661838	0.01338468	-4.9447408	7.78E-07	8011	NA	Nerve_Tibial	0.01171984	0.00417568	Nerve_Tibial
ENSG00000004700	RECQL	-0.2446941	0.04951561	-4.9417578	7.90E-07	8011	NA	Vagina	0.00577902	0.00417568	Vagina
ENSG00000272243	None	-0.1259842	0.02550608	-4.9393778	8.00E-07	8011	NA	Ovary	0.00699239	0.00417568	Ovary
ENSG00000140463	BBS4	-0.0954123	0.01935602	-4.9293329	8.42E-07	8011	NA	Thyroid	0.0123508	0.00429507	Thyroid; Uterus; Prostate; Small_Intestine_Terminal_Ileum; Ovary; Liver
ENSG00000104880	ARHGEF18	-0.1557729	0.0316743	-4.9179602	8.92E-07	8011	NA	Brain_Nucleus_accumbens_ basal_ganglia	0.00893072	0.00442899	Brain_Nucleus_accumbens_basal_ganglia
ENSG00000270820	USP34-DT	-0.0627886	0.01282113	-4.8972728	9.91E-07	8011	NA	Muscle_Skeletal	0.01087784	0.00446133	Muscle_Skeletal
ENSG00000090612	ZNF268	-0.2200657	0.04504655	-4.8852962	1.05E-06	8011	NA	Lung	0.01425548	0.00458499	Lung
ENSG00000166225	FRS2	0.07370449	0.01510025	4.88100977	1.08E-06	8011	NA	Whole_Blood	0.01111451	0.00464739	Whole_Blood
ENSG00000115839	RAB3GAP1	0.12712801	0.02606615	4.87713021	1.10E-06	8011	NA	Ovary	0.00959453	0.00470121	Ovary

ENSG00000121966	CXCR4	0.16333802	0.03368709	4.84868294	1.27E-06	8011	NA	Heart_Left_Ventricle	0.0143732	0.00525792	Heart_Left_Ventricle
ENSG00000162576	MXRA8	-0.2767463	0.0571321	-4.8439717	1.30E-06	8011	NA	Brain_Cerebellum	0.01525678	0.00525792	Brain_Cerebellum; Brain_Caudate_basal_ganglia; Liver; Brain_Putamen_basal_ganglia; Brain_Hypothalamus; Prostate; Brain_Cerebellar_Hemisphere; Artery_Coronary; Adrenal_Gland; Brain_Frontal_Cortex; Brain_Nucleus_accumbens_basal_ganglia; Brain_Hippocampus; Pancreas; Esophagus_Gastroesophageal_Junction; Heart_Atrial_Appendage; Brain_Amygdala; Brain_Cortex; Muscle_Skeletal
ENSG00000139946	PELI2	0.08847046	0.01827759	4.84037946	1.32E-06	8011	NA	Whole_Blood	0.01363861	0.00525792	Whole_Blood
ENSG00000135870	RC3H1	-0.2131424	0.04409394	-4.8338254	1.36E-06	8011	NA	Cells_EBV_transformed_ly mphocytes	0.00991552	0.00525792	Cells_EBV_transformed_lymphocytes; Brain_Cerebellar_Hemisphere
ENSG00000099953	MMP11	-0.0465763	0.00964472	-4.8292039	1.40E-06	8011	NA	Brain_Cerebellum	0.01642933	0.00525792	Brain_Cerebellum; Brain_Cerebellar_Hemisphere
ENSG00000186448	ZNF197	0.05375178	0.01115204	4.81990404	1.46E-06	8011	NA	Colon_Sigmoid	0.01804984	0.00525792	Colon_Sigmoid; Minor_Salivary_Gland
ENSG00000225285	LINC01770	0.79485154	0.16512885	4.8135232	1.51E-06	8011	NA	Lung	0.02044799	0.00525792	Lung
ENSG00000078674	PCM1	-0.0758111	0.01578739	-4.8020032	1.60E-06	8011	NA	Liver	0.01319793	0.0054127	Liver
ENSG00000165629	ATP5F1C	0.04313002	0.00900798	4.78797791	1.71E-06	8011	NA	Muscle_Skeletal	0.01882814	0.00552176	Muscle_Skeletal
ENSG00000146094	DOK3	0.0579086	0.01209675	4.78712057	1.72E-06	8011	NA	Adipose_Visceral	0.02266987	0.00552176	Adipose_Visceral; Adipose_Subcutaneous
ENSG00000258405	ZNF578	0.13314389	0.02792237	4.76835891	1.89E-06	8011	NA	Muscle_Skeletal	0.02075162	0.00568548	Muscle_Skeletal; Pituitary
ENSG00000144834	TAGLN3	-0.0698607	0.01465388	-4.7673835	1.90E-06	8011	NA	Pancreas	0.02077035	0.00568548	Pancreas; Heart_Atrial_Appendage
ENSG00000172403	SYNPO2	-0.1349189	0.02831576	-4.764801	1.92E-06	8011	NA	Adipose_Subcutaneous	0.02643331	0.00568548	Adipose_Subcutaneous; Adipose_Visceral
ENSG00000271736	LINC02772	-0.1021434	0.02143869	-4.7644413	1.93E-06	8011	NA	Testis	0.03219615	0.00568548	Testis
ENSG00000176105	YES1	0.08277323	0.01742467	4.75034606	2.07E-06	8011	NA	Esophagus_Muscularis	0.02784995	0.00587915	Esophagus_Muscularis; Esophagus_Gastroesophageal_Junction
ENSG00000261542	None	-0.4630366	0.09753777	-4.7472541	2.10E-06	8011	NA	Skin_sun_exposed_lower_le g	0.02940212	0.00589739	Skin_sun_exposed_lower_leg; Colon_Transverse; Prostate; Adrenal_Gland
ENSG00000124541	RRP36	0.10911386	0.02302244	4.73945653	2.18E-06	8011	NA	Prostate	0.02299988	0.00596405	Prostate
ENSG00000277763	None	-0.0610081	0.01289274	-4.7319718	2.26E-06	8011	NA	Cells_Cultured_fibroblasts	0.02887162	0.00596405	Cells_Cultured_fibroblasts; Brain_Hippocampus; Brain_Substantia_nigra
ENSG00000100554	ATP6V1D	0.07341853	0.01551999	4.73057792	2.28E-06	8011	NA	Adrenal_Gland	0.02379161	0.00596405	Adrenal_Gland; Pancreas; Stomach; Esophagus_Gastroesophageal_Junction; Breast_Mammary_tissue; Artery_Coronary; Liver; Ovary; Uterus
ENSG00000272416	None	-0.1740773	0.03681168	-4.7288598	2.30E-06	8011	NA	Prostate	0.02422976	0.00596405	Prostate
ENSG00000117601	SERPINC1	0.06496807	0.01375429	4.72347796	2.36E-06	8011	NA	Artery_Coronary	0.02401063	0.0060081	Artery_Coronary; Colon_Sigmoid
ENSG00000137285	TUBB2B	-0.0794211	0.01681924	-4.7220407	2.37E-06	8011	NA	Colon_Sigmoid	0.02930251	0.0060081	Colon_Sigmoid
ENSG00000146122	DAAM2	-0.0898263	0.01907899	-4.7081277	2.54E-06	8011	NA	Adipose_Subcutaneous	0.03493024	0.0062822	Adipose_Subcutaneous
ENSG00000026025	VIM	-0.1384532	0.02943299	-4.7040124	2.59E-06	8011	NA	Ovary	0.02268199	0.00632246	Ovary
ENSG00000102539	MLNR	-0.3574297	0.07598443	-4.7039867	2.59E-06	8011	NA	Testis	0.04333855	0.00632246	Testis
ENSG00000152128	TMEM163	-0.2266213	0.04824001	-4.6977879	2.67E-06	8011	NA	Artery_Tibial	0.03573498	0.00642834	Artery_Tibial; Brain_Anterior_cingulate_cortex; Prostate
ENSG00000136986	DERL1	0.21412494	0.04563716	4.69189835	2.75E-06	8011	NA	Prostate	0.02903328	0.00652712	Prostate
ENSG00000050344	NFE2L3	0.09805158	0.02094463	4.68146511	2.90E-06	8011	NA	Adrenal_Gland	0.03024857	0.00677661	Adrenal_Gland
ENSG00000276966	H4C5	-0.1476153	0.03166632	-4.6615876	3.19E-06	8011	NA	Stomach	0.03612944	0.00727116	Stomach; Whole_Blood
ENSG00000244968	LIFR-AS1	0.10341287	0.02220309	4.65758977	3.25E-06	8011	NA	Lung	0.04402053	0.00736281	Lung
ENSG00000132953	XPO4	-0.1063488	0.02287312	-4.6495092	3.38E-06	8011	NA	Whole_Blood	0.03492435	0.00745308	Whole_Blood; Pancreas; Cells_EBV_transformed_lymphocytes; Vagina; Brain_Amygdala
ENSG00000229117	RPL41	-0.2855312	0.06154662	-4.6392658	3.55E-06	8011	NA	Pancreas	0.03884475	0.00767195	Pancreas
ENSG00000116574	RHOU	0.12414074	0.02685226	4.62310236	3.84E-06	8011	NA	Heart_Left_Ventricle	0.04358389	0.00803202	Heart_Left_Ventricle
ENSG00000075131	TIPIN	-0.0756709	0.01637822	-4.6202168	3.89E-06	8011	NA	Cells_EBV_transformed_ly mphocytes	0.0282965	0.0080336	Cells_EBV_transformed_lymphocytes
ENSG00000175548	ALG10B	-0.1265743	0.02754133	-4.5957945	4.38E-06	8011	NA	Brain_Nucleus_accumbens_ basal_ganglia	0.04382731	0.0082775	Brain_Nucleus_accumbens_basal_ganglia; Brain_Anterior_cingulate_cortex
ENSG00000119917	IFIT3	-0.1189582	0.02592348	-4.5888195	4.53E-06	8011	NA	Brain_Cortex	0.04734978	0.0082775	Brain_Cortex
ENSG00000267940	None	-0.0797358	0.01739039	-4.5850499	4.61E-06	8011	NA	Minor_Salivary_Gland	0.03875494	0.0082775	Minor_Salivary_Gland
ENSG00000174446	SNAPC5	-0.0605362	0.01323767	-4.573022	4.88E-06	8011	NA	Cells_EBV_transformed_ly mphocytes	0.03546498	0.0082775	Cells_EBV_transformed_lymphocytes
ENSG00000249684	None	-0.1537816	0.03387676	-4.5394412	5.72E-06	8011	NA	Liver	0.04722373	0.0086876	Liver
ENSG00000231890	DARS1-AS1	-0.1291218	0.02856309	-4.5205826	6.26E-06	8011	NA	Vagina	0.04576578	0.0087931	Vagina
